# Extreme mito-nuclear discordance in a peninsular lizard: the role of drift, selection, and climate

**DOI:** 10.1038/s41437-019-0204-4

**Published:** 2019-03-04

**Authors:** Pedro Henrique Bernardo, Santiago Sánchez-Ramírez, Santiago J. Sánchez-Pacheco, Sergio Ticul Álvarez-Castañeda, Eduardo Felipe Aguilera-Miller, Fausto Roberto Mendez-de la Cruz, Robert W. Murphy

**Affiliations:** 1 Department of Ecology and Evolutionary Biology, University of Toronto, 25 Willcocks Street, Toronto, ON M5S 3B2 Canada; 2 Department of Natural History, Royal Ontario Museum, Toronto, ON Canada; 3 Centro de Investigaciones Biológicas del Noroeste, La Paz, Baja California Sur Mexico; 4 Laboratorio de Herpetología, Instituto de Biología, Universidad Nacional Autónoma de México, Mexico, Mexico

**Keywords:** Molecular evolution, Structural variation

## Abstract

Nuclear and mitochondrial genomes coexist within cells but are subject to different tempos and modes of evolution. Evolutionary forces such as drift, mutation, selection, and migration are expected to play fundamental roles in the origin and maintenance of diverged populations; however, divergence may lag between genomes subject to different modes of inheritance and functional specialization. Herein, we explore whole mitochondrial genome data and thousands of nuclear single nucleotide polymorphisms to evidence extreme mito-nuclear discordance in the small black-tailed brush lizard, *Urosaurus nigricaudus*, of the Peninsula of Baja California, Mexico and southern California, USA, and discuss potential drivers. Results show three deeply divergent mitochondrial lineages dating back to the later Miocene (ca. 5.5 Ma) and Pliocene (ca. 2.8 Ma) that likely followed geographic isolation due to trans-peninsular seaways. This contrasts with very low levels of genetic differentiation in nuclear loci (*F*_ST_ < 0.028) between mtDNA lineages. Analyses of protein-coding genes reveal substantial fixed variation between mitochondrial lineages, of which a significant portion comes from non-synonymous mutations. A mixture of drift and selection is likely responsible for the rise of these mtDNA groups, albeit with little evidence of marked differences in climatic niche space between them. Finally, future investigations can look further into the role that mito-nuclear incompatibilities and mating systems play in explaining contrasting nuclear gene flow.

## Introduction

Mitochondria are vital components of eukaryotic cells as they are directly involved in oxygen use, metabolism and energy production via oxidative phosphorylation (OXPHOS) (Saraste 1999). They have their own circular genome with 13 mitochondrial DNA (mtDNA) genes in vertebrates (Melo-Ferreira et al. 2014) that encode proteins in four of the five OXPHOS complexes (I, III, IV, and V) (Morales et al. 2015). Those genes work in conjunction with 72 nuclear DNA (nDNA) genes encoding proteins that participate in all five complexes to produce cellular energy (Saraste 1999; Morales et al. 2015; Wai and Langer 2016). The mtDNA genome has a higher rate of molecular evolution than nDNA and is inherited only from the female, usually without recombination with the male parent’s mtDNA. In contrast, nDNA is inherited biparentally and can recombine during meiosis.

Because all genes in the mitogenome are involved in energy production and metabolism, and consequently play vital roles in the organism’s life, the mtDNA genome is expected to evolve under purifying selection; deleterious mutations are selected against, so the functionality of the protein complexes remains unchanged (Ballard and Whitlock 2004; Pavlova et al. 2017). Although recent studies on fishes (Pavlova et al. 2017), birds (Morales et al. 2015), and mammals (Zhang et al. 2013) revealed widespread signatures of purifying selection in mtDNA genes, some codons have evolved under positive selection, which may indicate adaptation of specific lineages to physiological and environmental constraints (Yu et al. 2011; Morales et al. 2015; Pavlova et al. 2017; Jin et al. 2018).

Selection is expected to act in the same way on nDNA genes involved in the OXPHOS system to maintain compatibility with the mtDNA genome and preserve efficiency in energy production (Bar-Yaacov et al. 2015). This constrained coevolution of mtDNA and nDNA genes is known as mito-nuclear functional compensation. The strong functional link between these genes results in natural selection against hybrids, especially in zones of mtDNA discordance, to avoid individuals with inefficient energy production (Levin et al. 2014; Hill 2015). For years, scientists have been using mtDNA sequences to test evolutionary hypotheses of intraspecific relationships, species delimitation and phylogeny. More recently, however, nDNA markers not involved in the OXPHOS system (e.g., RAG exons, SNPs, some allozymes, and microsatellites) are being incorporated into molecular analyses. When a significant difference occurs between geographic patterns and genetic patterns of mtDNA and such nDNA markers, authors have hypothesized that mito-nuclear functional compensation maintains mtDNA discontinuities (Lindell et al. 2005, 2008; Yang and Kenagy 2009; Morales et al. 2015). Still, only very few studies have tested for mito-nuclear functional compensation when analyzing the nDNA genes directly involved in the OXPHOS system (Bar-Yaacov et al. 2015).

Species that present different geographic patterns between mtDNA and nDNA offer valuable opportunities to test the roles of neutrality and selection on mitogenomes (Morales et al. 2015), especially because selection on mtDNA genes can drive population divergence and speciation (Tieleman et al. 2009). One such species, the black-tailed brush lizard, *Urosaurus nigricaudus*, occurs on the Peninsular Ranges of Baja California, Mexico, and southern California, USA (Lindell et al. 2008). This common, xerophilic phrynosomatid is an arboreal species that can be found in a wide variety of natural environments as well as urban areas (Munguia-Vega et al. 2013). In its natural habitat, *U. nigricaudus* occupies Mesquite trees (*Prosopis palmeri*) and smaller shrubs in the “arroyos”, which are depressions in the landscape that form temporary rivers during the rainy season. Most of the environment on the peninsula consists of arid habitat, and the arroyos are the only areas that can hold water for a short period of time; making them the only areas where the trees that house *U. nigricaudus* occur, with the exception of oases.

**Fig. 1:**
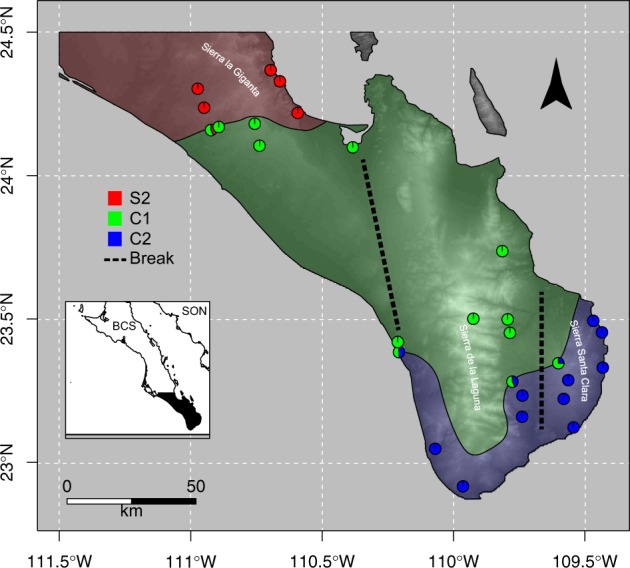
Map of southern Peninsula of Baja California, Mexico showing sampling localities. Dotted lines indicate possible seaway break locations. Points with mixed mtDNA lineages mark contact zone locations. Different shades represent the different mtDNA lineages: S2, C1, and C2. Map scale in kilometers. Source: Elevation data: Global Digital Elevation Model (DEM) from ASTER

*Urosaurus nigricaudus* has six parapatric mtDNA lineages that have substantial nucleotide sequence divergence (Lindell et al. 2008). After ephemeral isolation by seaways, populations of *U. nigricaudus* were reunited, yet female lineages remained distinct. The three most divergent mitochondrial lineages (S2, C1, and C2) occur in the Mexican state of Baja California Sur (Fig. 1). The deepest mtDNA divergence for *U. nigricaudus* occurs across the Isthmus of La Paz on the southern portion of the peninsula. Here, the mtDNA lineages (S2 vs. C1 + C2) differ by 11.02% in their sequences, and the second deepest discontinuity (C1 vs. C2) of 7.41% sequence divergence occurs further south in the region of Los Cabos (Lindell et al. 2008). In spite of these deep genealogical breaks, which far exceed most species divergences (Wu and Murphy 2015; Ivanov et al. 2018; Meik et al. 2018), no evidence of strong nDNA structure has been detected in either allozyme distributions (Aguirre-Léon et al. 1999) or analysis of thousands of nDNA SNPs (this study). Thus, analyses cannot reject the hypothesis of unrestricted nDNA gene flow (panmixia) throughout the species’ distribution.

Peninsular seaway breaks may explain how the mtDNA discontinuities arose, but a fundamental question remains unanswered: what mechanisms maintain mitochondrial lineages geographically restricted in the absence of physical barriers? The origin of these discordances date to millions of years ago, but no mechanistic explanation exists for their persistence. Herein, we use the mtDNA genome to investigate natural selection on mtDNA protein-coding genes and evaluate climatic niche divergence between mtDNA lineages. We contrast our results with evidence from SNP data of unrestricted nDNA gene flow and the time of origin of each of the mitochondrial lineages. We further discuss the roles that selection, climatic adaptation, and drift may play in the maintenance of mtDNA discontinuities, and comment on future research venues looking into how mito-nuclear functional compensation, behavior and sex-biased dispersal could contribute to these patterns.

## Materials and methods

### Sampling

During August 2013, 26 samples (Supplementary Table S1) of *U. nigricaudus* belonging to three distinct mtDNA lineages (S2, C1, and C2) were collected from several localities on the southernmost portion of the Peninsula of Baja California in Mexico. Sampling localities (Fig. 1) were chosen to maximize coverage of the lineages’ distributions. Lizards were collected by hand, photographed, measured and a small tissue sample was collected from the tail and preserved in 96% ethanol. All samples were collected in accordance with Animal Use Protocols by the Royal Ontario Museum Animal Care Committee and approved by Mexican authorities.

### MtDNA genome sequencing

All the laboratory work was done in the Laboratory of Molecular Systematics at the Royal Ontario Museum. The full mtDNA genome of *U. nigricaudus* was described by Bernardo et al. (2016). Polymerase chain reaction (PCR) amplifications were performed using universal mtDNA primers for squamate reptiles (Kumazawa and Endo 2004; Green et al. 2010) and specific primers for *U. nigricaudus* (Supplementary Table S2) were developed to amplify the remaining parts of the genome and to assure all sequences overlapped before assembly. Total genomic DNA was extracted from ethanol-preserved muscle tissue using the standard phenol-chloroform protocol proposed by Sambrook et al. (1989). The PCR mix (25 μl) contained: 18.55 μl of ddH_2_O, 2.5 μl of 1.5 mM MgCl_2_ buffer, 0.8 μl of 10 mM dNTPs, 1 μl of 10 mM of each primer, 0.15 μl of 5 U *Taq* DNA Polymerase (Boehringer Mannheim) and 1 μl (10 ng) of template DNA. Amplification reactions were performed on a Perkin Elmer GeneAmp 9700 and an Eppendorf AG 5345 thermal cycler (Applied Biosystems) using the follow program: initial denaturation of 94 °C for 2 min followed by 39 cycles of 94 °C for 30 s, 48–52 °C for 45 s, 72 °C 45 s, with a final extension temperature of 72 °C for 5 min. Amplified DNA was separated by electrophoresis on a 1% agarose gel and stained with SYBR Safe DNA Gel Stain (ThermoFisher). DNA bands were visualized on Safe Imager 2.0 Blue Light Transilluminator (ThermoFisher) and individually extracted and centrifuged through a filter pipette tip for 10 min at 3500 RPM. All samples were then sequenced for both directions (forward and reverse) using the BigDye Terminator v3.1 Cycle Sequencing Kit (Applied Biosystems). The sequencing reaction mix (10 μl) contained: 1 μl of BigDye, 2 μl of 5× BigDye Terminator Buffer, 2 μl of ddH_2_O, 1 μl of 10 mM of primer, and 4 μl of the PCR product. We then ran the sequencing reaction on an Eppendorf AG 5345 thermal cycler (Applied Biosystems) using the following program: initial denaturation at 96 °C for 1 min, 25 cycles of 96 °C for 10 s, 50 °C for 5 s, and 60 °C for 4 min. After the last cycle the temperature was set to 4 °C indefinitely. The reactions were then cleaned and precipitated with sodium acetate and ethanol and sequenced using a 3730 DNA Analyzer (Applied Bio-Systems).

The mitogenome assembly, gene annotation and alignments were done in geneious v11 (Kearse et al. 2012) using the mitogenome of the phrynosomatid *Sceloporus occidentalis* (GenBank Accession number AB079242) as a template.

### Mitochondrial divergence and genealogy reconstruction

Twenty-six mtDNA genomes were initially aligned using the muscle v3.8.31 plugin in geneious. For subsequent analyses, we only focused on the 13 protein-coding genes, which had a total alignment length of 10,833 bp. Depending on the analysis, we either concatenated gene partitions or used each alignment individually. Before analyses, we checked for recombination within our mitochondrial dataset using rdp4 (Martin et al. 2015) and excluded one individual sample that was found as a significant recombinant. We used paup* v4.0a161 (Swofford 2002) to calculate a sequence divergence matrix (uncorrected *p*-distances) for all samples using the mtDNA genome sequences.

To infer divergence times we used the Bayesian phylogenetic software beast v2.5 (Bouckaert et al. 2014). First, we estimated the substitution rate in *COI* by downloading available GenBank sequences of the family Phrynosomatidae, aligning them using mafft (Katoh and Standley 2013) and then building a maximum-likelihood phylogenetic tree with RAxML (Stamatakis 2014) (see Supplementary Figure S1). We then collapsed similar haplotypes into a single sequence per “species” cluster using mptp (Kapli et al. 2017). Those sequences were used to build a phylogenetic tree and estimate a global substitution rate in beast. This was done by applying a relaxed clock rate, a Yule speciation prior, and a calibration point at the root of the tree with a normal distribution prior (mean = 50 Ma, sd = 1.5) based on the age of Phrynosomatidae from TIMETREE (http://www.timetree.org/). One MCMC chain was run for 10 million generations sampling every 1000 states. The mean rate for *COI* was then used for the subsequent analysis with only the three mitochondrial lineages of *U. nigricaudus*.

To estimate divergence times in *U. nigricaudus*, one partition alignment for each of the 12 protein-coding genes was uploaded to beauti, where the tree prior was linked (i.e., concatenation) but the clock substitution models were unlinked across partitions. Substitution models for each gene partition were co-estimated throughout the analysis using bModelTest (Bouckaert and Drummond 2017), which uses Bayesian model averaging to infer and marginalize full time-reversible models. As a tree prior, we used a constant coalescent model and the default Jeffery’s (1/×) prior to estimate the population size parameter. As a clock model, we used a lognormally-distributed relaxed clock to allow for between-branch rate heterogeneity. We applied the previously estimated rate for *COI*, allowing the rate of the other genes to vary freely but scaled to the rate of *COI*. Two MCMC chains were run in parallel for a total of 50 million generations, sampling every 5000, to obtain a total of 10,000 posterior states. Parameter mixing and convergence was assessed in tracer v1.7, considering the analysis sufficiently sampled if ESS were higher or equal to 200, and convergence by comparing log files from parallel runs. treeannotator was used to generate a maximum-clade-credibility (MCC) tree after discarding 10% of states as burnin and applying the option “-heights ca”.

### Molecular evolution analyses

We looked at the molecular evolution of synonymous and non-synonymous sites using robust counting with beast v1.10 (Drummond et al. 2012; Lemey et al. 2012). This method draws power from the integration of stochastic character mapping and empirical Bayes to efficiently count synonymous and non-synonymous changes throughout the phylogeny, also considering the uncertainty in phylogeny estimation (Lemey et al. 2012). Alignments of all mitochondrial protein-coding genes were individually loaded to beauti, where we specified the codon-site-partitioned HKY model of nucleotide substitution, a lognormally-distributed relaxed clock model and selected the box for robust counting synonymous and non-synonymous mutations per branch. As priors, we selected constant coalescent as the tree model and fixed the ucld.mean parameter to 1 to have branch lengths in substitutions per site; all other parameters were defaulted. For this analysis, we were not interested in estimating divergence times, only in counting synonymous and non-synonymous changes. Analyses for each gene were run for 50 million generations, while sampling every 5000 states. We assessed convergence and parameter mixing by looking at the log file in tracer v1.7 (Rambaut et al. 2018), considering parameters with effective sample sizes (ESS) above 200 as sufficiently well sampled. Tree files were summarized using treeannotator (Drummond and Rambaut 2007) by removing a burnin of 1000 trees (from a total 10,000) and selecting the heights = ca option. The resulting MCC tree was processed in R using the package rBt (Sánchez-Ramírez 2018), where posterior metadata were extracted for the branch of each mtDNA lineage.

In addition, codon-based analyses were performed using codeml in the package paml v4.8a (Yang 1997, 2007). codeml used maximum likelihood to evaluate the codon substitution models and detect natural selection acting on protein-coding genes (Jeffares et al. 2015). For codeml, the models were divided into three classes: site models (*ω* can vary at different sites in the gene), branch models (*ω* can vary in different branches in a tree) and branch-site models (*ω* can vary in particular sites and in particular branches of the tree). We used the likelihood ratio test (LRT) to compare pairs of models (one model was set as a null model and the second model as the alternative model) and determine if the data had a significantly better fit to the alternative model.

The comparisons started with LRTs between the five different site models. First, null model M0 assumed one average *ω* for all sites and all lineages, then it was used in comparisons with alternative M1 model (nearly neutral model). This allowed for two site-classes with *ω* < 1 and *ω* = 1. If the LRT result was statistically significant, i.e., a non-neutral model fits better the data, then specific tests for positive selection were conducted. The LRT was then used to compare two pairs of models (Models 1 and 2a, and 7 and 8), where the null model did not allow for positive selection (*ω* < 1 and *ω* = 1) and the alternative model allowed for *ω* > 1. Null models M1 (nearly neutral model) and M7 (beta) allowed for *ω* < 1 and *ω* = 1. The alternative models M2a and 8, allowed for 0 ≤ *ω* < 1, *ω* = 1, and *ω* > 1. If the LRT results for the comparison of M1 × M2a and/or M7 × M8 were statistically significant, then some sites were assumed to be evolving under positive selection. To further investigate which of those sites might have been under positive selection, models M2a and M8 were used in a Bayes Empirical Bayes (BEB) analysis to calculate the Bayesian posterior probability of positively selected sites. Positively selected sites identified with this approach were considered to be statistically significant when the BEB posterior probability was greater than 0.95 and *ω* > 1 (Yang et al. 2005).

After completing the site model tests, we analyzed the branch models to test the null hypothesis that all lineages (branches in the mtDNA tree) were evolving under the same evolutionary rate. We used the LRT to compare model M0, which assumed that all lineages shared the same average *ω*, to the alternative model (two-ratio model) to check if a specific branch in the tree (foreground branch) had a *ω* value distinct from the other branches. We performed three LRT calculations selecting each one of the mitochondrial lineages (S2, C1, and C2) as the foreground branch to test if the evolutionary rate varied among the lineages. If the results were significant, we rejected the hypothesis that all mtDNA lineages are evolving under same *ω* and further tested those lineages for diversifying selection using the branch-site models.

For the branch-site models, we used the LRT to compare the null hypothesis that the selected mitochondrial lineage (foreground branch) was not evolving under positive selection using model A1 (0 < *ω* < 1; *ω* = 1), to the alternative hypothesis model A (0 < *ω* < 1; *ω* = 1; *ω* > 1), which allowed the foreground branch to be evolving under positive selection.

### Climatic niche divergence

To assess the role climate may have played in mitochondrial lineage divergence, 19 bioclimatic variables at a 30 arc seconds resolution (approx. 1 km^2^) were downloaded from WorldClim2 (http://worldclim.org/version2) and cropped to a binding box of −111.5, −108.5, 22.5, 24.5 decimal degrees. Coordinates in decimal degrees for 193 individuals were clustered using geographic distance [*rdist* function from the fields R package (Nychka et al. 2017)] and only a single sample was taken from clusters of samples within less than a radius of 500 m. This was done to remove redundancy in the climatic data due to close proximity. After this filtering step, we only used 36 sampled GPS coordinates (excluding two that were removed because no climatic data were associated with them). These coordinates were then used to extract environmental data from the raster layers of the 19 bioclimatic variables (*extract* function from the raster R package). Multiple linear regression and variable importance factors were used to determine multicolinearity, excluding highly colinear variables with theta ≥ 0.9. We then only focused on 11 variables (bio2, bio3, bio4, bio6, bio8, bio9, bio14, bio15, bio17, bio18, bio19; see http://worldclim.org/bioclim for details) for subsequent analyses. To collapse climatic multivariate data into a reduced set of ordinated dimensions, we performed a non-metric multidimensional scaling plot (*isoMDS* function from the R package mass). A total of five dimensions were evaluated as indicated by decreasing stress values. The dimension with the most discriminant power was then regressed against the bioclimatic variables using a standard least square method, followed by an estimation of relative variable importance using the “lmg” method. All analyses were done in R (R Core Team 2013) and figures were plotted with ggplot2 (Wickham 2016).

### SNP analysis

One hundred ninety-nine samples were processed for double-digest RAD-tag sequencing in the University of Arizona Genetics Core facility. The protocol followed was that of Peterson et al. (2012). The enzymes used were *Sph*I and *Mlu*CI. RAD-tag libraries for each individual were pooled into a single Illumina HiSeq 2500 lane using pair-ended chemistry at a length of 100 bp.

Raw sequence data were quality filtered by masking nucleotides with N’s if the phred quality was below 50. Then, we demultiplexed the reads into individual files using process_radtags from the stacks package v1.48 (Catchen et al. 2013). Reads were discarded if more than 50% Ns were found. We assembled RAD-tag loci de novo using usstacks with the following parameters: -M 6 -m 2 -H -d. A catalog of RAD-tag loci was built using cstacks and a maximum distance of 4 nucleotides (-n 4) to merge loci. We blacklisted loci from the catalog that were poorly represented and found no more than 20 times. Afterwards, each sample was re-matched to the catalog with sstacks. The program populations was used to generate a VCF file using the following parameters: -p 3 -r 0.2, which were meant to filter loci that were not found in at least 20% of the samples on all three mitochondrial populations.

To estimate *F*_ST_ values, we loaded the VCF file into R using the *read.vcf* function in the package pegas (Paradis 2010). We then converted that object into a “genind” object using the adegenet package (Jombart 2008; Jombart and Ahmed 2011), and calculated pairwise *F*_ST_ values with the *pairwise.fst* function in the package hierfstat (Goudet 2005). *F*_ST_ values were then converted into Nm values using the estimator from Hudson et al. (1992):

$$< {\mathrm{Nm}} > _{f_{{\mathrm{st}}}} = \frac{1}{4}\left( {\frac{1}{{F_{{\mathrm{ST}}}}} - 1} \right).$$

To visualize nDNA data, we computed a neighbor-joining tree using Euclidean distances between individuals based on multivariate genotype data. First, we used the R package gstudio (Dyer 2014) to compute the distances, and then the ape package (Paradis et al. 2004) to compute the neighbor-joining tree. In addition, we ordinated genetic data using non-metric multidimensional scaling (NMDS) using the function *isoMDS* from the mass package (Venables and Ripley 2002).

## Results

### Mitogenome tree and divergence times

**Fig. 2:**
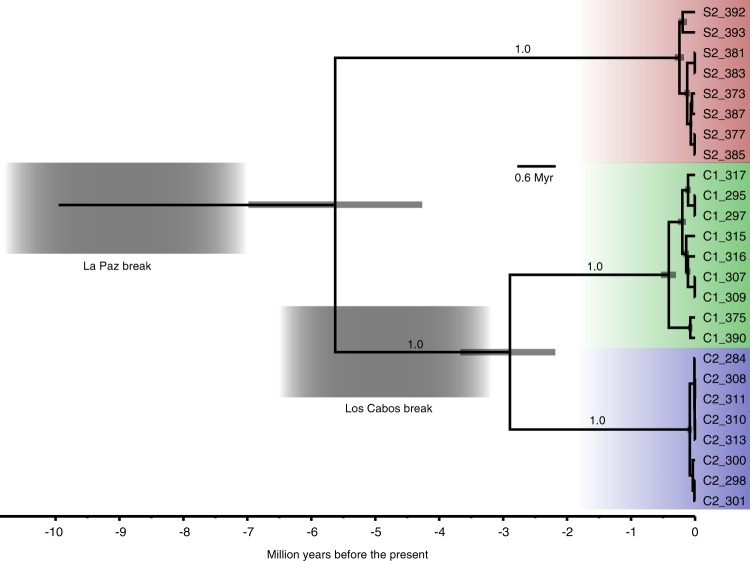
Mitogenome time-calibrated genealogical tree of *Urosaurus nigricaudus*. Gray node bars represent the 95% highest posterior density of the divergence time estimate. Thicker, translucent bars mark estimated times of seaway breaks based on geological data

Values of pairwise proportional sequence divergence (uncorrected *p-*distances) for the La Paz break ranged from 8.26 to 8.39% and for the Los Cabos Break from 5.23 to 5.41%. The substitution rate for the Phrynosomatidae was estimated at a mean of 0.0107 [HPD: 0.014, 0.0077] substitutions/site/Myr (Supplementary Figure S2). The time-calibrated mitogenome tree (Fig. 2) had very high support for the separation between each mtDNA lineage (posterior probability of 1.0). The divergence time for the split between S2 and C1 + C2 was estimated at mean of 5.62 [HPD 6.91, 4.26] Ma. For the split between C1 and C2, the mean divergence time was 2.89 [HPD 3.66, 2.18] Ma. In both cases, the estimated date of the seaway break predated the origin of the three mitochondrial lineages suggesting that divergence occurred after physical isolation and not before (Fig. 2).

### Molecular evolution analyses

**Fig. 3:**
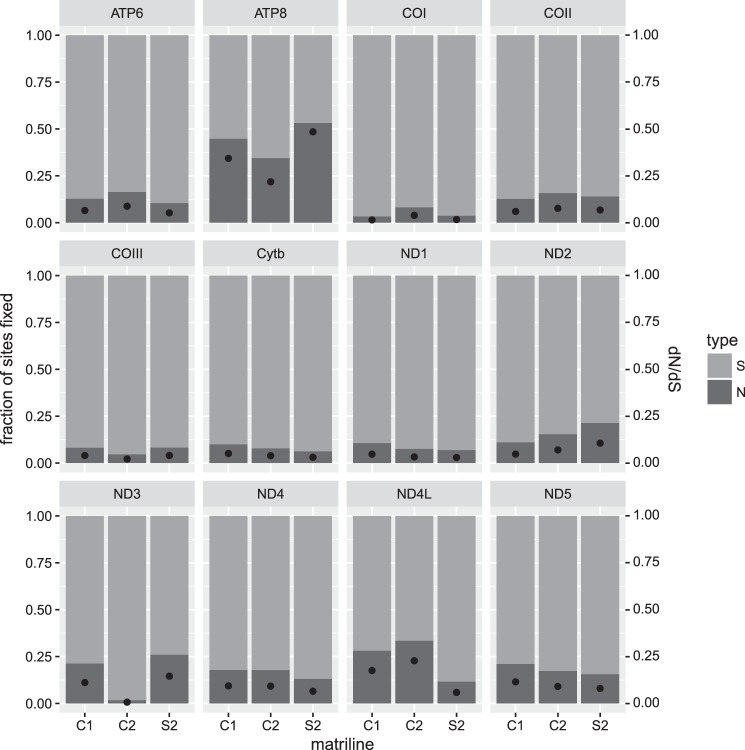
Molecular evolution on mitogenome protein-coding genes. Each barplot represents a gene. Each bar on the *x*-axis indicates one of three mtDNA lineages (S2, C1, C2). The left-side of the *y*-axis shows the proportion of fixed synonymous (S) and non-synonymous (N) mutations; the right-side displays the dN/dS ratio, which is marked by a black dot

Our first analysis using robust counting in beast indicated that a significant fraction of non-synonymous sites was fixed between the three mtDNA lineages (Fig. 3). However, mitochondrial protein-coding genes had mostly synonymous changes as fixed variants (Fig. 3). Lineage specific dN/dS ratios estimated for each mitochondrial lineage were consistent with codeml estimates (see below) and ranged from 0.0073 in *ND3* for C2 to 0.4846 in *ATP8* for S2 (Fig. 3). The gene with the most fixed non-synonymous sites was *ND2* in lineage S2, and the one with the least was *ND3* with 0 in lineage C2 (Table 1). Across all genes, 79 non-synonymous fixed mutations were found in lineage S2, followed by 50 in lineage C2, and 44 in lineage C1 (Table 1). Between 7.33 (S2) and 6.66 (C1 and C2) fixed synonymous sites occurred for every fixed non-synonymous site.

**Table 1:** Median number of synonymous (S) and non-synonymous (N) changes in mtDNA protein-coding genes fixed between mitochondrial lineages

	C1		C2		S2	
Gene	S	N	S	N	S	N
ATP6	16	2	22	4	44	5
ATP8	1	1	2	1	5	5
COI	50	2	47	4	92	3
COII	22	3	5	1	44	7
COIII	22	2	23	1	36	3
CYTB	44	5	52	4	87	6
ND1	17	2	31	2	51	4
ND2	42	5	14	2	66	18
ND3	7	2	5	0	11	4
ND4	28	6	65	14	94	14
ND4L	6	2	5	3	16	2
ND5	54	14	84	18	69	13
Total	293	44	333	50	571	79

The one ratio model (M0) in codeml estimated a mean *ω* = 0.033 for the 13 concatenated protein-coding genes, suggesting that overall the mtDNA genome was under strong purifying selection. *COI* had the highest level of purifying selection with *ω* = 0.006 and *ATP8* had the lowest with *ω* = 0.252.

The first analysis of site models found that *ND1*, *COI*, *COII*, *COIII*, *ND3*, *ND4L*, and *CYTB* were most likely to have one average *ω* for all codons in those genes (Supplementary Table S3). However, for *ND2*, *ATP8*, *ATP6*, *ND4*, *ND5*, and *ND6*, the best fit model was M1, which allowed some codons to evolve under different *ω* (Supplementary Table S3).

The test for positive selection on codons evolving under different values of ω (*ND2*, *ATP8*, *ATP6*, *ND4*, *ND5*, and *ND6*) suggested purifying or neutral selection. No significant signals of positive selection were detected (Supplementary Table S4). The BEB test suggested that some codons of *ATP8*, *ND2*, *ND4*, *ND5*, and *ND6* experienced positive selection, but none of those results were supported by Bayesian posterior probabilities (Supplementary Table S5).

Tests using the branch and branch-site models to evaluate if lineages were evolving independently under positive selection showed strong purifying selection (Supplementary Table S6); no signals of diversifying selection acting in the mtDNA lineages were detected (Supplementary Table S7). Purifying selection, however, seemed to have been relaxed in mitochondrial lineage S2. When branch S2 was set as the foreground branch for *ND1*, *ATP6*, *ND4*, *ND5*, and *Cytb*, there was significant evidence for S2 evolving under weaker purifying selection when compared to the other lineages. The values of branch *ω* for the mtDNA lineage S2 on those genes averaged 70% higher than the average *ω* for all lineages (*ω*_0;_ Supplementary Table S3).

### Climatic niche divergence

**Fig. 4:**
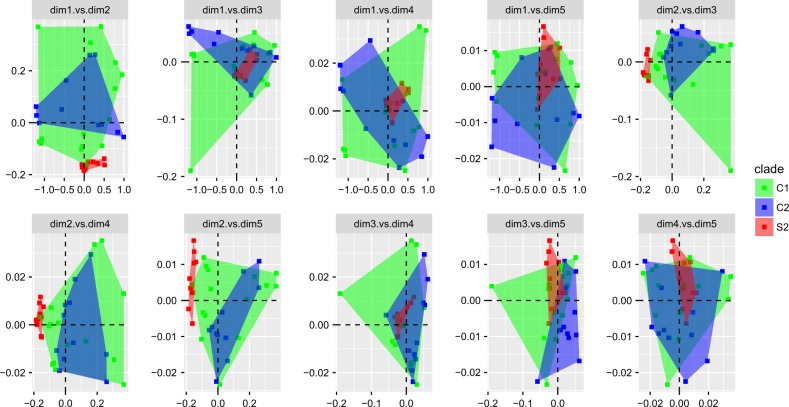
Climatic niche divergence between the different mtDNA lineages based on non-metric multidimensional scaling (NMDS). Each plot represents comparisons between five different dimensions in NMDS. Multivariate data are represented by 11 bioclimatic variables. Dimension 2 has the most discriminating resolution among the mtDNA lineages

Our multivariate analysis based on non-metric multidimensional scaling of 11 bioclimatic variables showed climatic divergence in only one dimension (dimension 2; Fig. 4), and only between S2 and C1 + C2. Mitochondrial lineages C1 and C2 overlapped broadly in their climatic niche-space (Fig. 4). Relative variable importance was found to be the highest in bioclimatic variables related to precipitation and less so in variables related to temperature (Supplementary Figure S3). Precipitation of the warmest quarter (bio18), precipitation of the driest quarter (bio17) and precipitation of the driest month (bio14) were among the highest variables.

### *F*_ST_ and gene flow in the nuclear genome

We analyzed a total of 15,351 SNPs for a total 199 individuals. The average coverage per SNP per individual was 5.28. Pairwise *F*_ST_ values ranged from 0.0186 between C1 and C2, to 0.0283 between S2 and C2 (Table 2). In addition, a neighbor-joining tree of genetic distances visualized the absence of strong population structure in the nuclear genome (Supplementary Figure S4). Thus, the SNP data did not reflect the three deeply divergent mtDNA lineages.

**Table 2:** Pairwise *F*_ST_ values between mtDNA lineages (lower diagonal) and Nm estimates (upper diagonal) based on 15,351 SNPs

	*C*1	*C*2	*S*2
*C*1		13.142	9.866
*C*2	0.0186		8.568
*S*2	0.0247	0.0283	

## Discussion

The mitochondrial and nuclear genomes are evolutionary semi-independent units. On the one hand, mtDNA is a compact circular segment that is uniparentally inherited, it usually does not recombine and it has faster rates of evolution than the nuclear genome. On the other hand, nDNA is biparentally inherited, recombines every generation and has a slower rate of molecular evolution than the mitogenome. However, mechanisms such as mito-nuclear functional compensation ties mitochondrial and nuclear OXPHOS machinery to the same evolutionary path (Levin et al. 2014; Hill 2015). Our analyses find high amounts of genetic divergence between the mitogenomes of three parapatric populations of *U. nigricaudus*. This genetic divergence is higher than divergence thresholds found between species (Hebert et al. 2004; Barrett and Hebert 2005; Poyarkov et al. 2017). While these parapatric mtDNA lineages are geographically structured and are likely to have originated via seaway breaks during the late Miocene and Pliocene (Figs. 1, 2), evidence from nDNA does not show the same population structure as the mtDNA data yield (Table 2 and Supplementary Figure S4).

### The role of selection, drift, and climate

Genes in the mitogenome are bound to the energy production process and, therefore, they evolve under strong purifying selection (Stewart et al. 2008). Our higher ratio of synonymous to non-synonymous polymorphism in mitochondrial protein-coding genes exemplifies this (Fig. 3; Table 1). While explicit tests of selection find no evidence that these genes have an excess of replacement variation compared to selectively neutral variation (Supplementary Tables S3–S7), analyses discover a high number of fixed non-synonymous variants between the different mitochondrial lineages (Fig. 3; Table 1). Simulations show that dN/dS > 1 are rarely obtained when there is strong selection within a population (Kryazhimskiy and Plotkin 2008). Thus, within a population, a single advantageous mutation is likely to drag along linked neutral and nearly neutral genetic variants (Barton 2000), which in the case of mtDNA would be the whole genome in the absence of frequent recombination. Given the natural overabundance of synonymous variation in the mitogenome, we expect that a large fraction of sites in this class will be fixed along with advantageous mutations, giving a signature of purifying selection (i.e., dN/dS < 1). Evolutionary forces such as drift and natural selection do not generally act in isolation, and their effect on the genome is largely dependent on population size (Ohta 1992; Tong et al. 2017). Population size reduction due to drift following isolation can also lead to an increase in frequency of effectively neutral non-synonymous variants (Popadin et al. 2007; Pavlova et al. 2017; Vasemägi et al. 2017).

Our time-calibrated phylogeny suggests that mtDNA lineage divergence occurred after allopatric barriers, in the form of trans-peninsular seaway breaks, took place (Figs. 1, 2). In isolated and small populations, drift plays a major role in genetic change and it can lead to the stochastic fixation of alleles. One possible scenario is that these events increased the genetic drift within the isolated populations in turn leading to high levels of divergence. Drift can also be more pronounced in mtDNA due to its lower effective population size compared to nDNA. However, we would expect to see effects on both genomes given the deep divergence times (Fig. 2). In contrast, the thousands of SNPs screened suggest high nuclear gene flow following secondary contact. Moreover, under drift, all fixed non-synonymous changes between mitochondrial lineages would need to be only slightly deleterious, which is rarely the case in mitochondrial genes (Stewart et al. 2008; Popadin et al. 2013). In this same line of reasoning, it is necessary to invoke philopatry or niche conservativism to explain the observed geographic structure. Some differences in climatic niche space may exist between S2 and C1 + C2, but not between C1 and C2 (Fig. 4). Further, mtDNA lineages extend across the putative original seaway barriers into secondary contact. This rejects the hypothesis of strong philopatry (Fig. 1; Table 2). More importantly, individuals of different mitochondrial lineages occur sympatrically in at least four arroyos (Fig. 1), and some are found only a few meters apart. This rejects the hypotheses of isolation by distance and geographic barriers. The sympatric mtDNA lineages share similar climatic requirements, and while we find some overall differences in niche space between S1 and C1 + C2 (Fig. 4; but not between C1 and C2), climatic requirements cannot possibly be different within contact zones (Fig. 1). This rejects the hypothesis that climate may drive the mitochondrial discordance that occurs commonly on the peninsula (Grismer 2002; Dolby et al. 2015).

### Future directions: mito-nuclear incompatibilities and mating systems

Strong selection on efficient respiration requires a certain degree of compatibility between OXPHOS genes in both genomes. However, different rates of evolution and inheritance modes can many times cause rapid build-up of genetic incompatibilities leading to incipient speciation by a mechanism known as mito-nuclear functional compensation (Bar-Yaacov et al. 2015; Hill 2015). Lindell et al. (2008) suggested that mito-nuclear functional compensation (Rand et al. 2004; Osada and Akashi 2011; van der Sluis et al. 2015; Sunnucks et al. 2017) maintained the mitochondrial lineages in *U. nigricaudus*. In this scenario, lineages that have diverged in allopatry come back into contact, but mtDNA introgression is selected against because it disrupts the strong functional link between mtDNA and OXPHOS-associated nDNA genes. Given the amount of evolutionary time that separates these mtDNA lineages, we would expect postzygotic isolation to be strong enough to lead to perceptible levels of structure in the nuclear genome, as has been shown in other systems (Palumbi 1994; Presgraves 2002; Nosil et al. 2005; Mavárez et al. 2006).

The chromosomal position of OXPHOS genes within the nuclear genomes also has important consequences for effective functioning of co-adapted genes (Hill 2015). In systems with gametic sex determination, X/Z-linked OXPHOS nuclear genes are predicted to have unequal effects between sexes due to Haldane’s rule (Orr 1997; Hill 2015). While the sex determination system (i.e., XX/XY vs. ZW/ZZ in females and males) in *U. nigricaudus* is unknown, the XX/XY system has been found to be widespread within Iguanids (Ezaz et al. 2009). For example, *Uta stansburiana* exhibits mitochondrial discordances (Upton and Murphy 1997) and yet it has the XX/XY system (Pennock et al. 1969). Thus, the mechanism of sex determination does not seem to play a role in its discordance, and the same may be true for *U. nigricaudus*.

Because the transmission of mtDNA is uniparental and that of nDNA is biparental, differences in behavior between males and females could also drive mito-nuclear discordance. While females of *U. nigricaudus* are extremely territorial and do not allow other females in their trees (P.H.B., pers. obs.), males may be able to disperse further away from contact zones allowing nuclear gene flow but restricting further mitochondrial inheritance. Although not many studies have been conducted on squamate reptiles, some show that sex-biased differences in behavior and fitness may reflect patterns of dispersal. For example, in *Lacerta*, Olsson et al. (1997) found that in both males and females, fitness was a strong component for dispersal and that males dispersed farther if relatedness was higher with respect to their neighbors. Similarly, Qi et al. (2013) found clear genetic evidence of male-biased dispersal and strong female phylopatry in *Phrynocephalus*. In terms of female behavior, levels of aggression are higher in females with higher levels of paternity acquisition in the social lizard *Egernia* (While et al. 2009). These findings suggest that both aggressive female territoriality and a higher propensity for dispersal in males may also contribute to discrepant divergence in *Urosaurus*.

In future research, finding patterns of divergence in nDNA OXPHOS genes matching those of mtDNA genes, as well as their physical chromosomal location, could help understand better the role of mito-nuclear incompatibilities. Furthermore, behavioral and genetic studies tracking sex-biased dispersal and aggression in and out of contact zones will help clarify the role of mating systems in the mito-nuclear discordance of *U. nigricaudus*.

## Conclusion

Three distinct mitochondrial lineages of *U. nigricaudus* are maintained in the southern range of the Peninsula of Baja California despite evidence of extensive nDNA gene flow. High divergence in mtDNA and very shallow genome-wide *F*_ST_ exemplify an extreme case for mito-nuclear discordance. We find substantial amounts of non-synonymous variants fixed between mtDNA lineages. This underlies the roles drift and selection played in the origin of these mitochondrial lineages; however, we cannot fully distinguish between these evolutionary forces. While some lineages show differences in climatic preferences, climate alone does not fully explain observed patterns. In summary, initial geographic isolation and drift are probably the drivers of high mitochondrial divergence, followed by nuclear gene flow, with climate adaptation playing a weak role, if any, in maintaining these lineages separate.

### Data archiving

The 26 mitochondrial DNA genomes of *Urosaurus nigricaudus*, with the exception of the control regions, are available under the GenBank accession numbers MH369811–MH369835. SNP and climatic data are available from the Dryad Digital Repository: 10.5061/dryad.1v1qf6f.

## Supplementary information

Supplementary figures.

Supplementary tables.
