# Supplementary material for: Extreme mito-nuclear discordance in a peninsular lizard: the role of drift, selection, and climate
Source: Heredity (Edinb). 2019 Mar 4;123(3):359–70. doi: 10.1038/s41437-019-0204-4 (PMC6781153; doi:10.1038/s41437-019-0204-4)
Supplement: Supplementary file 1 — Supplementary figures. [file 41437_2019_204_MOESM1_ESM.docx]

**Extreme mito-nuclear discordance in a peninsular lizard: the role of drift, selection and climate**

Pedro Henrique Bernardo, Santiago Sánchez-Ramirez, Santiago J. Sánchez-Pacheco, Sergio Ticul Álvarez-Castañeda, Eduardo Felipe Aguilera-Miller, Fausto Roberto Mendez-de la Cruz, Robert W. Murphy

**SUPPLEMENTARY FIGURES**

**Supplementary Fig. S1.** Maximum-likelihood phylogenetic tree of cytochrome oxidase I (*COI*) sequences belonging to the subfamily Phrynosomatinae available in GenBank.


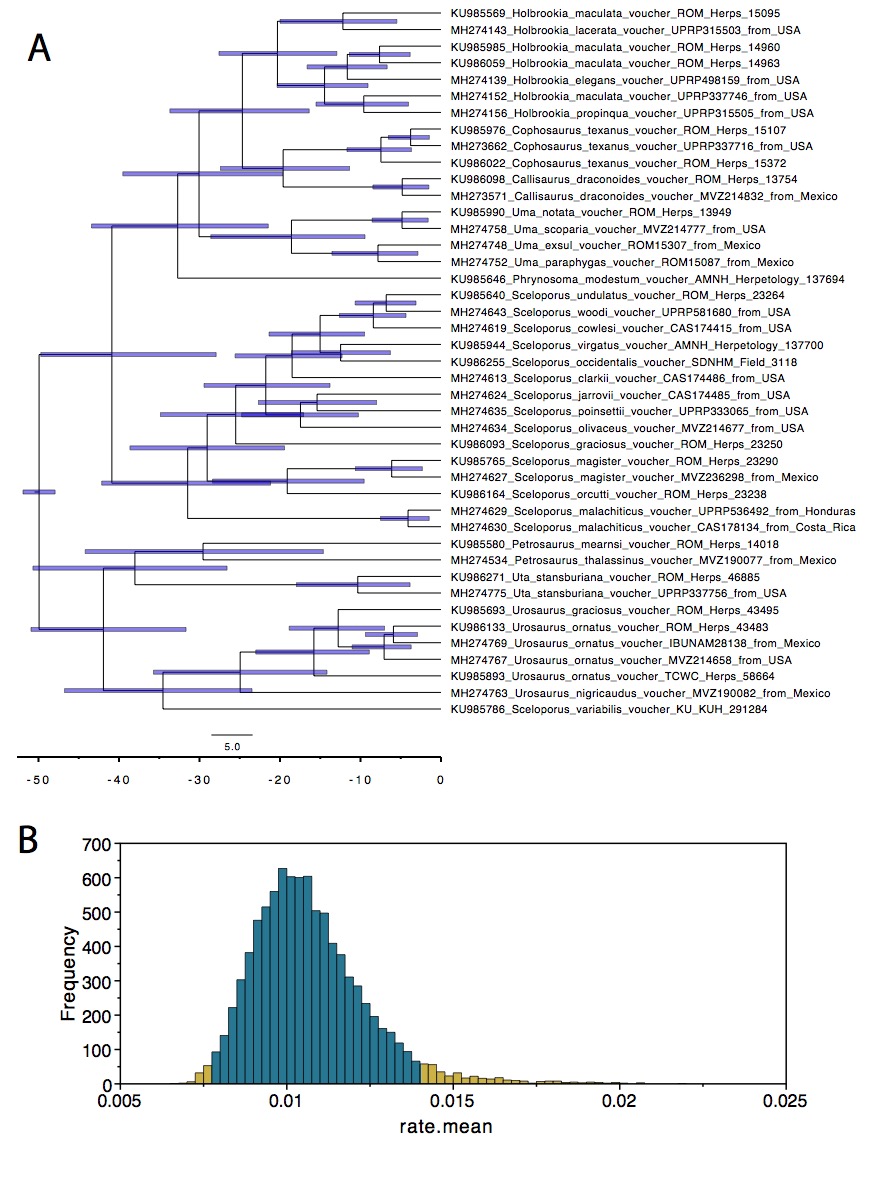


**Supplementary Fig. S2.** (A) Species-level time-calibrated phylogeny of the subfamily Phrynosomatinae inferred using beast; and (B) marginal posterior estimates of the substitution rate of cytochrome oxidase I (*COI*).

**Supplementary Fig. S3.** The proportion of relative variable importance for 11 bioclimatic variables based on a multiple linear regression of the variables on the dimension 2 of a non-metric multidimensional scaling plot analysis.

**Supplementary Fig. S4.** (A) Neighbor-joining tree of genetic distances of *Urosaurus nigricaudus* based on 15,351 single nucleotide polymorphisms; and (B) ordinated genetic data based on a two-dimension non-metric multidimensional scaling plot analysis. Individuals belonging to one of three mtDNA lineages (S2, C1, C2) are color marked.
