# Supplementary material for: Extreme mito-nuclear discordance in a peninsular lizard: the role of drift, selection, and climate
Source: Heredity (Edinb). 2019 Mar 4;123(3):359–70. doi: 10.1038/s41437-019-0204-4 (PMC6781153; doi:10.1038/s41437-019-0204-4)
Supplement: Supplementary file 2 — Supplementary tables. [file 41437_2019_204_MOESM2_ESM.docx]

**Extreme mito-nuclear discordance in a peninsular lizard: the role of drift, selection and climate**

Pedro Henrique Bernardo, Santiago Sánchez-Ramirez, Santiago J. Sánchez-Pacheco, Sergio Ticul Álvarez-Castañeda, Eduardo Felipe Aguilera-Miller, Fausto Roberto Mendez-de la Cruz, Robert W. Murphy

**SUPPLEMENTARY TABLES**

Supplementary Table S1 Samples of *Urosaurus nigricaudus* used in this study. ROM voucher represents the deposit number of the tissue in the herpetological collection of the Royal Ontario Museum.

| mtDNA lineage | Sample ID | ROM Voucher | Locality | LAT | LONG |
| --- | --- | --- | --- | --- | --- |
| S2 | 392 | 54671 | Km 20 N of La Paz towards San Juan de La Costa | 24.218388 | -110.59275 |
| S2 | 393 | 54774 | Km 85 N of La Paz on Hwy 1 towards Ciudad Constitución | 24.228201 | -110.9473 |
| S2 | 373 | 54642 | Km 77 N of La Paz on Hwy 1 towards Ciudad Constitución | 24.162766 | -110.92177 |
| S2 | 377 | 54775 | Km 77 N of La Paz on Hwy 1 towards Ciudad Constitución | 24.163484 | -110.92153 |
| S2 | 381 | 54770 | Km 77 N of La Paz on Hwy 1 towards Ciudad Constitución | 24.163523 | -110.92134 |
| S2 | 383 | 54644 | Km 77 N of La Paz on Hwy 1 towards Ciudad Constitución | 24.163544 | -110.9215 |
| S2 | 385 | 54641 | Km 77 N of La Paz on Hwy 1 towards Ciudad Constitución | 24.163571 | -110.92178 |
| S2 | 387 | 54769 | Km 77 N of La Paz on Hwy 1 towards Ciudad Constitución | 24.164033 | -110.92114 |
| S2 | 394 | 54773 | Km 86.5 N of La Paz on Hwy 1 towards Ciudad Constitución | 24.235923 | -110.94821 |
| C2 | 284 | 54739 | Oasis San Pedrito - Pescaderos | 23.388902 | -110.20822 |
| C2 | 298 | 54629 | Oasis San Pedrito - Pescaderos | 23.389067 | -110.20893 |
| C2 | 300 | 54632 | Oasis San Pedrito - Pescaderos | 23.389075 | -110.20861 |
| C2 | 308 | 54777 | Oasis San Pedrito - Pescaderos | 23.38919 | -110.21105 |
| C2 | 310 | 54750 | Oasis San Pedrito - Pescaderos | 23.389234 | -110.21111 |
| C2 | 311 | 54778 | Oasis San Pedrito - Pescaderos | 23.389566 | -110.21114 |
| C2 | 313 | 54779 | Oasis San Pedrito - Pescaderos | 23.389632 | -110.21124 |
| C2 | 301 | 54784 | Oasis San Pedrito - Pescaderos | 23.389076 | -110.20891 |
| C1 | 297 | 53168 | Oasis San Pedrito - Pescaderos | 23.389066 | -110.20902 |
| C1 | 375 | 54765 | Km 77 N of La Paz on Hwy 1 towards Ciudad Constitución | 24.163388 | -110.92161 |
| C1 | 390 | 54704 | Km 58 N of La Paz on Hwy 1 towards Ciudad Constitución | 24.18078 | -110.75625 |
| C1 | 295 | 54743 | Oasis San Pedrito - Pescaderos | 23.389057 | -110.20942 |
| C1 | 307 | 54776 | Oasis San Pedrito - Pescaderos | 23.389032 | -110.2088 |
| C1 | 309 | 54749 | Oasis San Pedrito - Pescaderos | 23.389234 | -110.21111 |
| C1 | 315 | 54783 | Oasis San Pedrito - Pescaderos | 23.389846 | -110.21137 |
| C1 | 316 | 54780 | Oasis San Pedrito - Pescaderos | 23.389922 | -110.21141 |
| C1 | 317 | 54781 | Oasis San Pedrito - Pescaderos | 23.389922 | -110.21141 |

Supplementary Table S2 List of the 22 pairs of primers used to sequence the complete mitochondrial genome of *Urosaurus nigricaudus.* Pair ID was used for lab work and to illustrate the position of the primers in the mtDNA genome (see Figure 1). Authors: 1 – This study; 2 – Green *et al*. (2010); 3 – Kumazawa and Endo (2004).

| Pair ID | FORWARD | | REVERSE | | Author |
| --- | --- | --- | --- | --- | --- |
|  | **Primer name** | **Sequence** | **Primer name** | **Sequence** |  |
| 1 | 12S1L | CCAACTGGGATTAGATACCCCACTAT | 16S-3H | GTAGCTCACTTGATT TCGGG | 2 |
| 2 | 12S2LM | ACACACCGCCCGTCACCCT | 16S2H | CCGGATCCCCGGCCGGTCTGAACTCAGATCACG | 2 |
| 3 | 16S_ND1F1 | AGTAAAACTGATCACCGAACC | 16S_ND1R2 | ATGTCAGTTATTGCGTGTGG | 1 |
| 4 | ND1_ND2F2 | ATGCCTATGACATACAGCCC | ND1_ND2R1 | TCTTCTAGGATTAGTCATTTTGG | 1 |
| 5 | ND2-COIF2 | CTCAAACACGAAAAATCATAGCC | ND2-COIR1 | GAAATGATGGGGGTAGCAG | 1 |
| 6 | COI-ATP6F2 | ACAGACCGMAACCTAAACAC | COI-ATP6R2 | GGGGTTTAGTTGTGGCATRTCACTG | 1 |
| 7 | COII-ATP6F1 | GCCCTACCATCCCTACGAATCC | COII-ATP6R1 | TAGTACTGTTGCTAGTCATATTGG | 1 |
| 8 | uLys-lL | AGCACTAGCCTTTTAAGC | uCO3-1H | AAYGTCTCGTCATCATTG | 3 |
| 9 | uCO3-1L | ATAGTWGACCCMAGCCCATGACC | uND3-2H | GGGTCRAAKCCRCATTCRTA | 3 |
| 10 | uCO3-3L | GAAGCMGCWGCCTGATACTGACA | rND4L-2H | GCTAGGCCAGTRCYTGCTTCRCA | 3 |
| 11 | rND4L-1L | TGCATTGAARGYATAATACT | uND4-2H | CTACRTGKGCTTTTGGKARTCA | 3 |
| 12 | rND4L-2L | TAACCTTCTCMGCMTGYGAAGC | rND4-2H | GATGTTAAKCCGTGGGCRATTAT | 3 |
| 13 | rND4-3L | CCAAAAGCCCAYGTAGARGC (20) | rCUN-1H | CTTTTACTTGGADTTGCACC | 3 |
| 14 | rHis-2L | AACAAAAACAYTAGRCTGTG | rND5-1H | ACWACTATTGTGCTKGAGTG | 3 |
| 15 | rND5-1L | TCCAAGCMATYATCTAYAACCG | rND5-2H | ATWGYGTCTTTTGAGTARAAKCC | 3 |
| 16 | rND5-2L | GAACARGACATYCGAAAAATRGG | rND6-4H | ATGTTAGTGGTDTTTGCKTATTC | 3 |
| 17 | rND5-3L | YACMYMAACGCCTGAGCCCT | rGlu-1H | ATTACAACGGYGGTTTTTC | 3 |
| 18 | rND6-3L | GCAACWGAATAHGCAAATAC | ucytb-1H | GCCCCTCAGAATGATATTTGTCCTCA | 3 |
| 19 | rGlu-1L | GAAAAACCRCCGTTGTWATTCAACTA | rcytb-1H | GCGTAGGCRAATAGGAAGTATCA | 3 |
| 20 | rcytb-2L | TGAGGACAAATATCMTTCTGAGG | rPro-1H | TTAAAATKCTAGTTTTGG | 3 |
| 21 | rThr-2L | YAAAGCMTTGRTCTTGTAA | rCONT-4H | CTCGKTTTWGGGGTTTGRCGA | 3 |
| 22 | rCONT-4L | TCGYCAAACCCCWAAAMCGAG | r12S-1H | TRTAACCGCGGTKGCTGGCAC | 3 |

**Supplementary Table S3** Results of the LRT comparison to evaluate whether the codons evolved with one average ω (null model M0 - ln*L*_0_) or with different values of ω (alternative Model M1- ln*L*_1_). ω_0_ represents null model M0. Level of significance determined by *p*-value with degree of freedom k = 1.

| Gene | ω_0_ | lnL_0_ | lnL_1_ | 2△lnL | P-value |
| --- | --- | --- | --- | --- | --- |
| ND1 | 0.025 | -1835.808407 | -1835.508491 | 0.6 | P > 0.05 |
| **ND2** | **0.042** | **-2067.001802** | **-2055.605821** | **22.79** | **P < 0.001** |
| COI | 0.006 | -2949.845390 | -2949.846805 | 0.003 | P > 0.05 |
| COII | 0.021 | -1309.726386 | -1309.607986 | 0.24 | P > 0.05 |
| **ATP8** | **0.252** | **-312.274257** | **-309.151277** | **6.24** | **P < 0.05** |
| **ATP6** | **0.038** | **-1358.593554** | **-1350.028209** | **17.2** | **P < 0.001** |
| COIII | 0.025 | -1493.681866 | -1493.682508 | 0.001 | P > 0.05 |
| ND3 | 0.055 | -587.588976 | -585.434041 | 4.31 | P > 0.05 |
| ND4L | 0.030 | -566.289713 | -564.741847 | 3.09 | P > 0.05 |
| **ND4** | **0.050** | **-2812.126577** | **-2800.476474** | **23.3** | **P < 0.001** |
| **ND5** | **0.047** | **-3908.761557** | **-3881.472377** | **54.58** | **P < 0.001** |
| **ND6** | **0.037** | **-958.607197** | **-955.473377** | **6.26** | **P < 0.05** |
| CYTB | 0.017 | -2416.027053 | -2414.211442 | 3.63 | P > 0.05 |

**Supplementary Table S4** Results of the statistical tests of adaptive evolution among codon sites using site models. LRT comparisons between models M1 x M2a and M7 and M8. Significant level determined by *p*-value with degree of freedom k = 2.

| **Gene** | **Null model** | **lnL_0_** | **Alternative Model** | **lnL_1_** | **2△lnL** | **P-value** |
| --- | --- | --- | --- | --- | --- | --- |
| **ND2** | M1 | -2055.605821 | M2a | -2055.392457 | 0.42 | P > 0.05 |
|  | M7 | -2057.471946 | M8 | -2055.329498 | 4.28 | P > 0.05 |
| **ATP8** | M1 | -309.151277 | M2a | -308.878294 | 0.54 | P > 0.05 |
|  | M7 | -309.177614 | M8 | -308.878317 | 0.59 | P > 0.05 |
| **ATP6** | M1 | -1350.028209 | M2a | -1350.028209 | 0 | P > 0.05 |
|  | M7 | -1350.253373 | M8 | -1350.034204 | 0 | P > 0.05 |
| **ND4** | M1 | -2800.476474 | M2a | -2800.152466 | 0.64 | P > 0.05 |
|  | M7 | -2799.412841 | M8 | -2799.732305 | 0.63 | P > 0.05 |
| **ND5** | M1 | -3881.472377 | M2a | -3881.472376 | 0 | P > 0.05 |
|  | M7 | -3879.311547 | M8 | -3878.619375 | 1.4 | P > 0.05 |
| **ND6** | M1 | -955.473377 | M2a | -955.473376 | 0 | P > 0.05 |
|  | M7 | -955.275532 | M8 | -955.275532 | 0 | P > 0.05 |

**Supplementary Table S5** Results of the Bayes Empirical Bayes (BEB) analysis calculated using Models M2a and M8 (beta and *ω*) to identify positively selected sites. BEB posterior probability that ω > 1 (Pr) was considered significant if greater than 0.95.

| **Gene** | **Site Number** | **Amino acid** | **M2a**  **Pr** | **M8**  **Pr** |
| --- | --- | --- | --- | --- |
| **ATP8** | 46 | H | 0.744 | 0.852 |
| **ND2** | 91 | D | 0.531 | 0.680 |
|  | 153 | T | - | 0.533 |
|  | 237 | S | 0.821 | 0.930 |
|  | 328 | S | 0.669 | 0.753 |
| **ND4** | 28 | M | - | 0.569 |
|  | 55 | Q | 0.511 | 0.614 |
|  | 139 | N | 0.572 | 0.704 |
|  | 189 | N | 0.567 | 0.696 |
|  | 198 | I | 0.514 | 0.614 |
|  | 424 | I | - | 0.511 |
|  | 426 | A | - | 0.507 |
| **ND5** | 206 | D | 0.648 | 0.843 |
|  | 267 | S | - | 0.570 |
|  | 361 | S | 0.508 | 0.616 |
|  | 365 | F | 0.746 | 0.934 |
|  | 368 | T | - | 0.530 |
|  | 467 | L | 0.595 | 0.761 |
|  | 493 | T | - | 0.512 |
|  | 573 | M | - | 0.544 |
|  | 597 | L | - | 0.511 |
| **ND6** | 89 | I | 0.505 | 0.587 |

**Supplementary Table S6** Results of the statistical tests of ω variation among lineages using **Branch** models. LRT results are from the comparison between null model M0, which assumes the same ω for lineages, and the alternative Two-Ratio model that assumes a different ω for a specific branch on the tree (foreground branch). Significant level determined by *p*-value with degree of freedom k = 1.

| **Gene** | **Branch** | **lnL** | | **Branch ω** | | **2△lnL** | **P-value** | |  |
| --- | --- | --- | --- | --- | --- | --- | --- | --- | --- |
| ND1 | **S2** | **-1832.574462** | | **0.042** | | **6.47** | **P < 0.05** | |  |
|  | C1 | -1835.434993 | | 0.026 | | 0.74 | P > 0.05 | |  |
|  | C2 | -1835.625141 | | 0.028 | | 0.37 | P > 0.05 | |  |
| ND2 | S2 | -2067.001952 | | 0.042 | | 0.003 | P > 0.05 | |  |
|  | C1 | -2066.902327 | | 0.042 | | 0.20 | P > 0.05 | |  |
|  | C2 | -2066.740045 | | 0.044 | | 0.52 | P > 0.05 | |  |
| COI | S2 | -2949.847209 | | 0.006 | | 0.004 | P > 0.05 | |  |
|  | C1 | -2949.719705 | | 0.006 | | 1.49 | P > 0.05 | |  |
|  | C2 | -2949.100122 | | 0.004 | | 0.25 | P > 0.05 | |  |
| COII | S2 | -1308.825223 | | 0.034 | | 1.80 | P > 0.05 | |  |
|  | C1 | -1309.074574 | | 0.020 | | 1.30 | P > 0.05 | |  |
|  | C2 | -1310.013374 | | 0.021 | | 0.57 | P > 0.05 | |  |
| ATP8 | S2 | -311.918388 | | 0.359 | | 0.003 | P > 0.05 | |  |
|  | C1 | -312.274245 | | 0.252 | | 0.20 | P > 0.05 | |  |
|  | C2 | -312.238750 | | 0.271 | | 0.52 | P > 0.05 | |  |
| ATP6 | **S2** | **-1355.770202** | | **0.067** | | **5.64** | **P < 0.05** | |  |
|  | C1 | -1357.786488 | | 0.038 | | 1.6 | P > 0.05 | |  |
|  | C2 | -1358.512343 | | 0.038 | | 0.160 | P > 0.05 | |  |
| COIII | S2 | -1492.024275 | | 0.036 | | 3.31 | P > 0.05 | |  |
|  | C1 | -1493.465164 | | 0.023 | | 0.43 | P > 0.05 | |  |
|  | C2 | -1493.406589 | | 0.027 | | 0.55 | P > 0.05 | |  |
| ND3 | S2 | -586.772772 | | 0.029 | | 1.63 | P > 0.05 | |  |
|  | C1 | -587.588975 | | 0.055 | | 0 | P > 0.05 | |  |
|  | C2 | -586.342571 | | 0.070 | | 2.49 | P > 0.05 | |  |
| ND4L | S2 | -566.289797 | | 0.030 | | 0 | P > 0.05 | |  |
|  | C1 | -566.030100 | | 0.032 | | 0.52 | P > 0.05 | |  |
|  | C2 | -565.579814 | | 0.035 | | 1.41 | P > 0.05 | |  |
| ND4 | **S2** | **-2807.623388** | | **0.075** | | **9** | **P < 0.05** | |  |
|  | C1 | -2811.829158 | | 0.050 | | 0.6 | P > 0.05 | |  |
|  | C2 | -2812.119693 | | 0.050 | | 0.01 | P > 0.05 | |  |
| ND5 | **S2** | **-3906.301764** | | **0.087** | | **4.91** | **P < 0.05** | |  |
|  | C1 | -3908.761519 | | 0.047 | | 0 | P > 0.05 | |  |
|  | C2 | -3908.759494 | | 0.047 | | 0.004 | P > 0.05 | |  |
| ND6 | S2 | -958.29489 | | 0.029 | | 3.7 | P > 0.05 | |  |
|  | C1 | -958.564032 | | 0.037 | | 0.8 | P > 0.05 | |  |
|  | C2 | -956.709038 | | 0.046 | | 0.62 | P > 0.05 | |  |
| CYTB | | **S2** | **-2409.038050** | | **0.032** | **13.98** | | **P < 0.001** | |
|  |  | C1 | -2414.791526 | | 0.015 | 2.47 | | P > 0.05 | |
|  |  | C2 | -2415.712382 | | 0.015 | 0.62 | | P > 0.05 | |

**Supplementary Table S7** Results of the statistical tests of adaptive evolution among sites and lineages using Branch-site models. LRTs were used to compare null model A1 (**ln*L*_0_**), which does not allow the specific branch on the tree (foreground branch) to evolve under positive selection to alternative model A (**ln*L*_1_**), which allows for positive selection.

| **Gene** | **Branch** | **lnL_0_** | **lnL_1_** | **2△lnL** | **P value** |
| --- | --- | --- | --- | --- | --- |
| ND1 | S2 | - 1835.509016 | - 1835.508491 | 0.001 | P>0.05 |
| ATP6 | S2 | -1350.028385 | -1350.028209 | 0 | P>0.05 |
| ND4 | S2 | -2800.477283 | - 2800.476474 | 0.002 | P>0.05 |
| ND5 | S2 | -3881.472486 | -3881.472375 | 0.001 | P>0.05 |
| CYTB | S2 | -2414.211442 | -2414.212615 | 0.002 | P>0.05 |
|  | C1 | -2412.931690 | -2412.931690 | 0 | P>0.05 |
